# Predictive diagnostic value of mean platelet volume to platelet count and neutrophil to lymphocyte ratios in the gray zone of prostate cancer with tPSA between 4 to 10 ng/mL

**DOI:** 10.3389/fonc.2024.1454124

**Published:** 2024-10-14

**Authors:** Xinyu Yi, Jin Li, Yilin Li, Tao Huang, Baiyi Xiong, Feng Zhang, Zhaoyi Zhao

**Affiliations:** ^1^ Xiangtan Central Hospital, Xiangtan, Hunan, China; ^2^ Nanchang University, Nanchang, Jiangxi, China

**Keywords:** PVI, NLR (neutrophil-to-lymphocyte ratio), prostate cancer, tPSA gray zone, predictive

## Abstract

**Objective:**

Exploration of the Predictive Diagnostic Value of Mean Platelet Volume to Platelet Count Ratio (MPV/PLT,PVI) and Neutrophil-to-Lymphocyte Ratio (NLR) in the tPSA Gray Zone of Prostate Cancer

**Methods:**

A retrospective study was conducted on 65 prostate cancer (Pca) patients and 52 benign prostatic hyperplasia (BPH) patients who underwent transperineal prostate biopsy at Xiangtan Central Hospital from December 2021 to December 2023. Descriptive statistics and logistic regression models were used to investigate the predictive diagnostic value of PVI and NLR in the tPSA gray zone of prostate cancer. Receiver operating characteristic (ROC) curves were constructed based on PVI and NLR values to determine the classification thresholds.

**Results:**

A total of 117 patients were enrolled, including 65 cases of prostate cancer (PCa) and 52 cases of benign prostatic hyperplasia (BPH). There were no statistically significant differences in age, BMI, history of hypertension, history of diabetes, history of coronary heart disease, pre-biopsy white blood cell count, history of drinking, history of smoking, and tPSA between the PCa and BPH patients. The results of logistic regression analysis showed that PVI (OR=2.03, 95%CI: 1.34~3.07, *P*<0.00) and NLR (OR=0.32, 95%CI: 0.18~0.58, *P*<0.00) were independent predictors for diagnosing prostate cancer in the tPSA gray zone (VIF=1.04).The maximum area under the curve (AUC) for PVI was 0.70, with an optimal cut-off value of 0.05 *(P ≤* 0.01). The maximum AUC for NLR was 0.76, with an optimal cut-off value of 2.86 (*P ≤* 0.01).The calibration curve showed good consistency between the predicted and actual outcomes in both the PCa and BPH groups, indicating that the nomogram model had good predictive performance.When using PVI and NLR to plot the receiver operating characteristic (ROC) curves to predict the assessment of PCa in the tpsa gray zone, the area under the curve (AUC) for PVI was the largest at 0.70, with an optimal cutoff value of 0.05 (*P* ≤ 0.01). The AUC for NLR was the largest at 0.76, with an optimal cutoff value of 2.86 (*P* ≤ 0.01).

**Conclusion:**

PVI and NLR have certain predictive diagnostic value for Pca in the tPSA gray zone, and appropriate use of PVI and NLR can improve the positive rate of early screening for Pca in the gray zone.

## 1 Background

Prostate cancer mainly occurs in middle-aged men aged 45-60, and is one of the most common malignant tumors affecting men worldwide, posing a significant health burden globally (1). Prostate cancer can generally be diagnosed through prostate biopsy, tPSA testing, digital rectal exam, MRI, etc. Androgen deprivation therapy is the main treatment for advanced prostate cancer (2). Early prostate cancer often has no obvious symptoms, so it is frequently diagnosed at a later stage, which creates major challenges in treatment. Therefore, early diagnosis of prostate cancer is of great significance for urologists in managing this disease.

Prostate-specific antigen (tPSA) is a glycoprotein secreted by prostate epithelial cells and is one of the most commonly used tools for prostate cancer screening. The commonly used cutoff is 4 ng/mL, and the tPSA gray zone is defined as 4-10 ng/mL. Patients in this gray zone have about a 25% chance of having prostate cancer, while tPSA >10 ng/mL has over 50% probability of prostate cancer. However, tPSA is not highly specific, as it can also be elevated in benign prostatic hyperplasia, prostatitis, and after prostate procedures. Nevertheless, tPSA remains the most commonly used screening test for prostate cancer (3, 4).

Mean platelet volume (MPV) is an early activation indicator of platelets, reflecting their structure and function (5). Some studies have shown MPV is associated with various cancers (6). Activated platelets (PLT) also serve as a systemic inflammation marker and participate in cancer development, progression and metastasis (7). The PVI ratio combines the characteristics of both, providing a new approach for prostate cancer prediction. Similarly, the neutrophil-to-lymphocyte ratio (NLR) has been found to be higher in cancer patients compared to healthy individuals, and is considered an independent predictor for certain cancers, including prostate cancer (8).

Therefore, this study aims to investigate the diagnostic value of PVI and NLR for prostate cancer prediction in the tPSA gray zone.

## 2 Materials and methods

The Xiangtan Central Hospital is the strongest comprehensive tertiary hospital in Xiangtan City. The urology department is a city-level clinical key specialty in Hunan Province. We conducted a retrospective study on 65 patients with prostate cancer (Pca) and 52 patients with benign prostatic hyperplasia (BPH) who underwent transperineal prostate biopsy at the Xiangtan Central Hospital from December 2021 to December 2023.

Inclusion criteria: 1) Underwent transperineal prostate biopsy in our hospital; 2) tPSA was between 4-10 ng/mL; 3) Complete case information.

Exclusion criteria: 1) Inflammatory diseases such as pneumonia, urinary tract infection, prostatitis; 2) Indwelling catheter before biopsy; 3) Accompanied by autoimmune diseases; 4)Accompanied by thrombosis or coagulation dysfunction.

Biopsy method: The patient was placed in the lithotomy position, routinely disinfected, the scrotum was pulled upwards, and the biopsy point was 2 cm above the anal canal and 1.5 cm lateral to the midline. One needle was punctured at the basal and body segments of the left and right lobes of the prostate, and two needles were punctured on the left and right peripheral lines, for a total of 10-12 needles. The biopsy specimens were collected, the tail ends were stained to distinguish them from the head ends, and then placed in a specimen bottle containing 10% formalin for pathological examination.

All patients were divided into the BPH group (control group) and the Pca group (observation group) according to the biopsy results. Patients’ general clinical data, including age, BMI, hypertension, diabetes, coronary heart disease, smoking history, and drinking history, were also collected. All patients underwent blood sampling for tPSA detection and blood routine examination within 1 week before prostate biopsy. The neutrophil-to-lymphocyte ratio (NLR) was calculated based on the neutrophil and lymphocyte values in the blood routine report, and the mean platelet volume/platelet count (PVI) ratio was also collected.

SPSS26.0 was used for descriptive statistics and multivariate Logistic regression analysis to explore the diagnostic value of PVI ratio and NLR in distinguishing BPH and Pca. The R(4.3.2) software was used to plot the receiver operating characteristic (ROC) curve and calculate the area under the curve (AUC) to diagnose BPH and Pca, as well as the optimal diagnostic threshold. *P ≤* 0.01 was considered statistically significant.

## 3 Results

The body mass index (BMI) of the 117 patients was 22.03 kg/m2. The white blood cell count before biopsy was 7.65 × 10^9/L. The tPSA was 6.29 ng/mL. 46 patients (39%) had hypertension, 20 patients (17%) had coronary heart disease, and 15 patients (13%) had diabetes. 34 patients (29%) had a history of drinking, and 94 patients (80%) had a history of smoking.

Among the 117 patients, 65 (55.6%) had Pca, and compared to the 52 (44.4%) BPH patients, the Pca patients had a lower PVI ratio (*P* ≤0.01), and their NLR was higher than the BPH patients (*P* ≤0.01). The two groups were similar in terms of hypertension, coronary heart disease, diabetes, BMI, pre-biopsy blood white blood cell count, drinking history, smoking history, and tPSA (**Table 1**).

**Table 1:** Comparison of various indicators between the PCa group and the BPH group (No. = 117).

	Pca	BPH	*p*
No. of patients (No)	65	52	
Age (years)	64.51 ± 8.26	69.58 ± 7.06	0.17
BMI (kg/m2)	21.99 ± 23.96	22.09 ± 2.92	0.74
Hypertension (No)	24	22	0.55
Coronary Heart Disease (No)	12	8	0.66
Diabetes Mellitus (No)	9	6	0.71
White blood cells count (× 10^9^/L)	7.89 ± 2.03	7.35 ± 2.09	0.09
Smoking history(No)	54	40	0.41
Alcohol history (No)	21	12	0.39
Tpsa (ng/mL)	6.52 ± 1.86	6.12 ± 1.59	0.10
PVI	0.04 ± 0.01	0.05 ± 0.02	0.00
NLR	3.35 ± 0.56	2.67 ± 0.81	0.00

As shown in **Table 1**, PVI is associated with prostate cancer (PCa) in the tPSA gray zone (P ≤ 0.01). From **Table 2**, we can see that:PVI (OR=2.03, 95%CI: 1.34~3.07, P<0.00) is an independent predictor for diagnosing PCa in the tPSA gray zone. NLR (OR=0.32, 95%CI: 0.18~0.58, P<0.00) is also an independent predictor for diagnosing PCa in the tPSA gray zone.

**Table 2:** The odds ratios of PVI and NLR.

	*p*	OR	95% confidence interval	
			upper limit	lower limit
PVI	0.00	2.03	1.34	3.07
NLR	0.00	0.32	0.18	0.58

**Figure 1:**
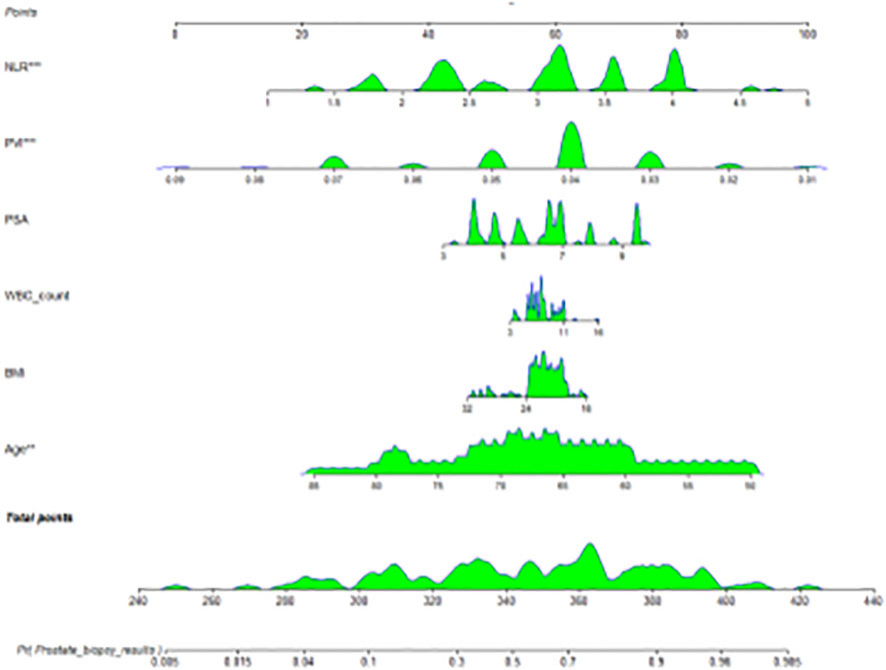
A nomogram for predicting prostate cancer.

**Figure 2:**
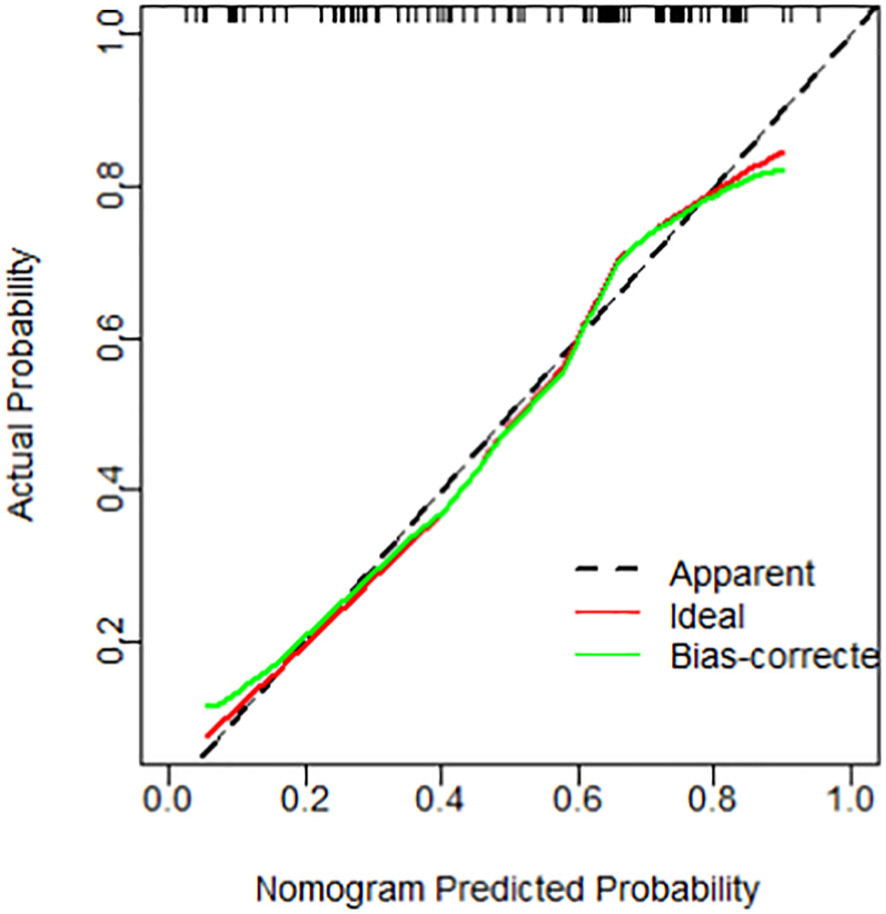
A calibration curve for predicting prostate cancer.

Further analysis revealed that PVI and NLR are independent predictors for diagnosing prostate cancer in the tPSA gray zone (VIF=1.04). A nomogram was constructed to predict prostate cancer in the tPSA gray zone, incorporating 6 variables: PVI, NLR, PSA, WBC count, BMI, and Age (**Figure 1**). The calibration curves for both the PCa group and the BPH group showed good alignment between the predicted probabilities and the actual outcomes (**Figure 2**), indicating that the nomogram model had good predictive performance. This suggests that the nomogram, which includes PVI and NLR as key predictors, can be a useful tool for accurately diagnosing prostate cancer in patients with tPSA levels in the gray zone.

**Figure 3:**
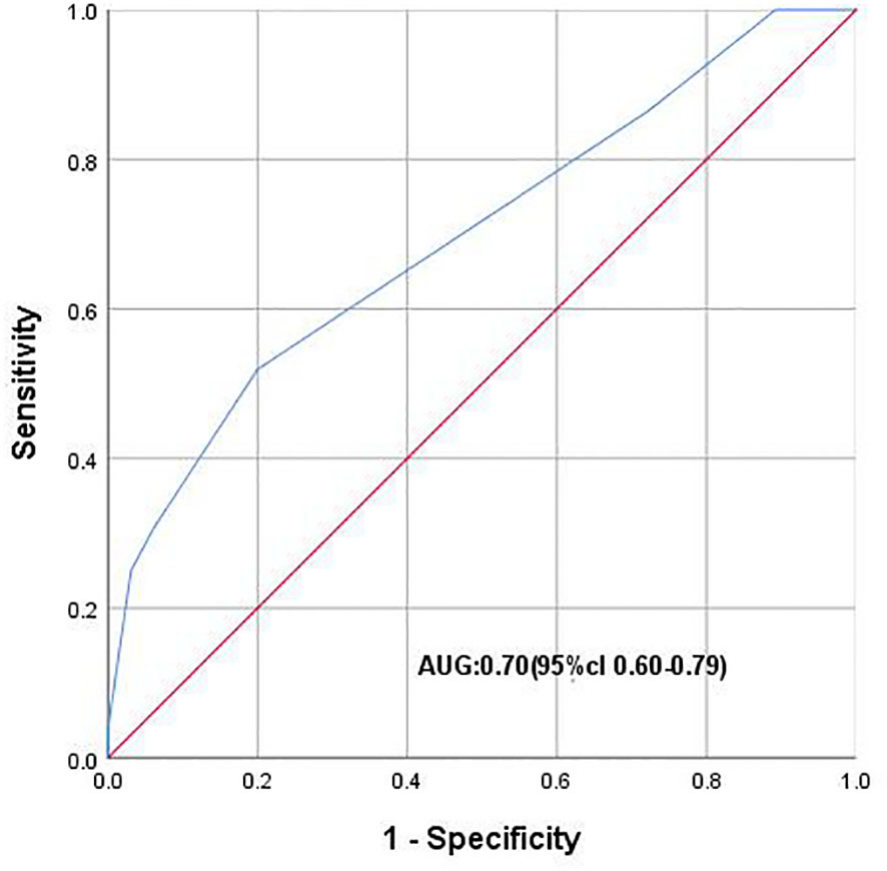
ROC curve plotting 1-speciffcity (x axis) against sensitivity (y axis). AUC, Area under the curve.

PVI was associated with Pca in the gray zone of tPSA (*P* ≤ 0.01). ROC analysis showed that PVI could distinguish Pca and BPH in the tPSA gray zone (AUC 0.70; 95% CI: 0.60-0.79). When the Youden index was 0.32, the optimal cutoff value of PVI was 0.05. The PVI cutoff value of 0.05 could diagnose the presence of PCa in the tPSA gray zone (**Figure 3**).

**Figure 4:**
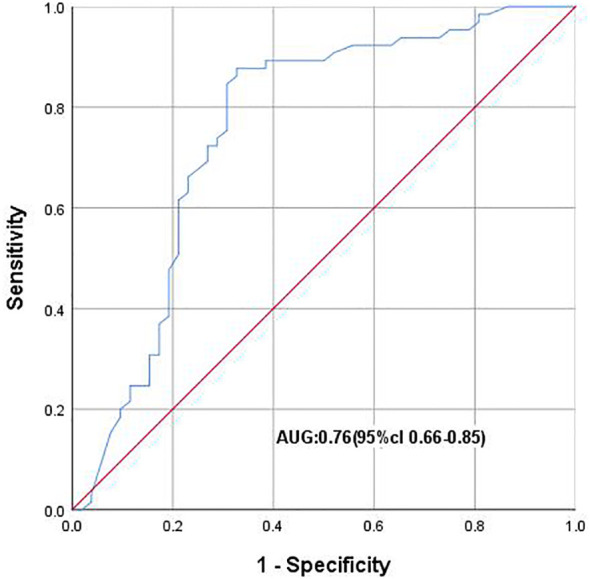
ROC curve plotting 1-speciffcity (x axis) against sensitivity (y axis). AUC, Area under the curve.

As shown in **Table 1**, the NLR was associated with Pca in the tPSA gray zone (*P* ≤ 0.01). ROC analysis showed that NLR could distinguish Pca and BPH in the tPSA gray zone (AUC 0.76; 95% CI: 0.66-0.85). When the Youden index was 0.21, the optimal cutoff value of NLR was 2.86. The NLR cutoff value of 2.86 could diagnose the presence of PCa in the tPSA gray zone (**Figure 4**).

## 4 Discussion

Prostate cancer (Pca) is the second most common malignant tumor in men globally and the second leading cause of cancer-related deaths, especially in developed countries (9). Pca is considered an extremely heterogeneous malignancy. The diagnosis, prognosis, and treatment of Pca have become complex due to this high degree of heterogeneity. The early screening of Pca still mainly relies on monitoring patients’ PSA levels, but this approach has certain limitations, especially when PSA values are in the 4-10 ng/mL range, where the predictive effect is poor. For Pca treatment, although the long-term survival rate of Pca is not low, late-stage Pca remains largely incurable even after intensified multimodal therapy, so early diagnosis of Pca patients is particularly important.

Inflammation-related neutrophils and immune cells (including lymphocytes) have been found to be associated with tumor formation (10). Inflammation plays an important role in the formation and development of tumors by promoting angiogenesis, proliferation, and protecting tumors from apoptosis. Tumor cells can attract pro-inflammatory cells to the tumor microenvironment by secreting various chemoattractants (11). Among these inflammatory parameters reflecting the systemic inflammatory response, the PVI ratio has been studied repeatedly, and it has been found to be significantly lower in patients with bone malignancies compared to normal individuals, and is associated with poor prognosis in patients (12). Some scholars have also found that the PVI ratio is decreased in breast cancer patients compared to normal individuals (13), but there are currently no studies on the role and impact of PVI in Pca. Therefore, we chose PVI as the research object to analyze its role and impact in Pca. Meanwhile, the increase in the neutrophil-to-lymphocyte ratio (NLR) has been found to be valuable in predicting the clinical outcomes of cancer patients. NLR is obtained from routine blood tests. Numerous studies have shown that in various malignant tumors such as gastric cancer, colorectal cancer, breast cancer, prostate cancer, soft tissue sarcoma, and non-small cell lung cancer, an increase in pretreatment NLR is associated with poor prognosis (14, 15).

Previous studies by Kormiluk A et al. showed that the PVI value in Pca patients was lower than in BPH patients (6), but relatively few studies have focused on Pca in the tPSA gray zone, i.e., the PSA range of 4-10 ng/mL. Our study found that the PVI value can predict Pca in the tPSA gray zone (*P* ≤ 0.01), and the PVI cutoff value of 0.05 can diagnose the presence of Pca in the tPSA gray zone. This provides another reference indicator for whether to perform prostate biopsy in the PSA gray zone.

Studies have shown that the inflammatory response plays an important role in the occurrence and development of tumors, and the immune response in tumor patients is closely related to lymphocytes (16). As a tumor predictive factor, NLR has certain predictive value for the prognosis of Pca patients. Systematic analysis of multiple studies has summarized that by stratifying the population into low NLR and high NLR groups, and using the Youden index to determine the cutoff value through receiver operating characteristic curve analysis, the optimal cutoff values of NLR vary from 1.7 to 5.0 in different studies (17). Based on the support of previous studies, we calculated the NLR values of the two groups and found a significant difference between the Pca group and the BPH group (*P* ≤ 0.01), and the NLR value of Pca patients was higher than that of BPH patients. The NLR cutoff value of 2.86 can diagnose the presence of Pca in the tPSA gray zone, which is consistent with the latest research.

In summary, our study to some extent demonstrates the predictive and diagnostic value of PVI and NLR for Pca in the tPSA gray zone, and appropriate use of PVI and NLR can improve the positive rate of early Pca screening. However, this study was a single-center retrospective study with a narrow patient selection range, and local differences in diet, culture, habits, climate, and living conditions may have introduced biases in the results. Additionally, the limited sample size and the significant influence of patient-specific factors on the experimental results require expanding the sample size and population range in future studies to eliminate these interfering factors. Despite strict case selection, there may still be unknown factors influencing the experimental results due to the limitations of clinical implementation and conditions. Future studies should aim to exclude other interfering factors as much as possible and conduct prospective studies.

## Data Availability

The raw data supporting the conclusions of this article will be made available by the authors, without undue reservation.
